# Impact of Anticoagulant Class on Long-Term Bioprosthesis Durability Following Transcatheter Aortic Valve Replacement

**DOI:** 10.1016/j.shj.2025.100786

**Published:** 2025-12-13

**Authors:** Antonin Trimaille, Pablo Vidal-Cales, Carlos Giuliani, Juan Hernando Del Portillo, Jean-Michel Paradis, Siamak Mohammadi, Anthony Poulin, Frederic Beaupré, Eric Dumont, Jean Porterie, Erwan Salaun, Marisa Avvedimento, Josep Rodés-Cabau

**Affiliations:** Quebec Heart & Lung Institute, Laval University, Quebec City, Quebec, Canada

**Keywords:** Anticoagulation, Direct oral anticoagulant, Durability, Hemodynamic valve deterioration, Transcatheter aortic valve replacement, Vitamin K antagonist

## Abstract

**Background:**

Vitamin K antagonists (VKAs) and direct oral anticoagulants (DOACs) have distinct biological properties that may differentially influence bioprosthetic valve durability following transcatheter aortic valve replacement. The aim of this study was to explore the effect of oral anticoagulation (OAC) class on bioprosthetic valve durability.

**Methods:**

We analyzed the data of a prospective registry including 688 consecutive patients under OAC undergoing transcatheter aortic valve replacement between May 2007 and January 2024 who were alive at 1 year. The effect of OAC class was assessed using a propensity score-matched population (132 patients with VKA vs. 132 patients with DOAC). The primary endpoint was the occurrence of stage 2 or 3 hemodynamic valve deterioration according to Valve Academic Research Consortium-3 criteria.

**Results:**

In the propensity score-matched population, treatment with DOACs was not associated with a different risk of stage 2 or 3 hemodynamic valve deterioration compared to VKAs (subdistribution hazard ratio [sHR] 0.89; 95% CI 0.35-2.29; *p* = 0.808) after a median follow-up of 4 years (interquartile range: 3-5). No significant differences were observed for the risk of bioprosthetic valve failure (sHR 1.62; 95% CI 0.53-4.96; *p* = 0.401) or aortic valve reintervention (sHR 0.97; 95% CI 0.14-6.82; *p* = 0.981). Long-term echocardiographic follow-up showed similar evolution of hemodynamic parameters over time.

**Conclusions:**

No significant differences were observed between VKAs and DOACs on valve durability outcomes. Further studies with longer follow-up, larger population, and randomized designs are warranted to confirm these findings.

## Highlights

• Vitamin K antagonists and direct oral anticoagulants have distinct biological profiles that may differently affect bioprosthetic valve durability after transcatheter aortic valve replacement.
• This study evaluated the long-term impact of oral anticoagulation class on bioprosthetic valve durability.
• After a median follow-up of 4 years, treatment with direct oral anticoagulants was not associated with a different risk of stage 2 or 3 hemodynamic valve deterioration, bioprosthetic valve failure, or aortic valve reintervention compared with vitamin K antagonists.
• Longitudinal echocardiographic follow-up demonstrated comparable trends in valve hemodynamics over time between the 2 groups.
• Further large-scale, randomized studies with extended follow-up are warranted to confirm these results.

## Introduction

The long-term durability of bioprosthetic valves has emerged as a major concern in patients undergoing transcatheter aortic valve replacement (TAVR), particularly as indications extend to younger, lower-risk populations with longer life expectancy.^1^^,^^2^ In this context, optimizing valve performance requires the identification of modifiable factors that enhance the risk of valve dysfunction.

Among these factors, growing evidence involves leaflet thrombosis as a potential contributor to bioprosthetic valve dysfunction.^3^ Both imaging and histopathological studies suggest that leaflet thrombosis may initiate structural valve deterioration through a mechanism of thrombus-mediated calcification.^4^, ^5^, ^6^ Several procedural and anatomical characteristics inherent to TAVR, such as valve underexpansion or asymmetric deployment,^7^ and persistent interaction with the diseased native aortic valve,^5^^,^^8^ may predispose transcatheter valves to thrombosis and ultimately impair long-term durability.

The role of oral anticoagulation (OAC) in the prevention of bioprosthetic valve dysfunction remains debated. Although some studies have reported that OAC is associated with improved valve hemodynamics and reduced incidence of valve dysfunction,^9^, ^10^, ^11^ others have suggested a paradoxical association between long-term OAC use and increased risk of structural deterioration.^12^ One potential explanation for these conflicting findings lies in the pharmacologic differences between anticoagulant classes. Although vitamin K antagonists (VKAs) may accelerate bioprosthetic valve calcification by inhibiting matrix Gla protein, a key regulator of vascular and valvular mineralization,^13^, ^14^, ^15^ direct oral anticoagulants (DOACs) may protect the bioprosthesis from the deleterious elevated level of Factor Xa in patients with severe aortic stenosis.^16^ To date, no study has assessed the long-term impact of anticoagulant class on transcatheter heart valve performance. Therefore, the present study aimed to evaluate the association between OAC class and long-term bioprosthetic valve durability following TAVR.

## Methods

### Study Design and Population

This observational study is based on a single-center prospective registry enrolling all consecutive patients undergoing TAVR at the Québec Heart and Lung Institute (Québec City, Canada). The indications for TAVR, device type, and procedural approach were assessed by the local heart team based on a comprehensive clinical and anatomic preoperative assessment. Transfemoral access was used by default, and alternative access in patients with unfavorable peripheral anatomy. Either balloon-expandable (Edwards Sapien, Sapien XT, Sapien 3 or Sapien 3 Ultra) or self-expanding valves (Medtronic CoreValve, Evolut FX, Evolut R and Evolut Pro; Boston Accurate and Accurate Neo2; and Abbott Portico) were used, according to clinical and anatomic characteristics. Prosthesis sizes ranged from 20 to 34 ​mm and were selected based on pre-TAVR echocardiography or cardiac computed tomography measurements. The registry was approved by the local ethics committee, and all patients provided written informed consent for the procedures. The study was conducted in compliance with the Declaration of Helsinki.

Only patients under OAC at discharge from the hospitalization for the TAVR procedure were included. The study included consecutive patients undergoing TAVR between May 11, 2007, and January 1, 2024. Those patients who died within the first year were excluded in order to better capture long-term outcomes and minimize attrition bias. Patients underwent clinical and echocardiographic follow-up with a minimum of 1 follow-up echocardiography ≥12 months from the date of TAVR.

The population was stratified based on the class of OAC prescribed at discharge: VKA (warfarin) or DOAC (apixaban, rivaroxaban, edoxaban, or dabigatran).

### Data Collection and Study End Points

Baseline, procedural, and follow-up data were prospectively collected in a dedicated TAVR data set. Clinical follow-up was performed during prescheduled outpatient clinic visits or by phone contact at 1-3 months, 12 months post-TAVR, and yearly thereafter. The vital status of the patient was updated after every medical contact, recording the date of the last contact for every patient. Additional information was obtained, when necessary, by consulting records from referring cardiologists, general practitioners, and other hospitals. All adverse events were prospectively collected and defined based on the Valve Academic Research Consortium (VARC)-3 definitions.^3^

Echocardiographic follow-up was performed at discharge and at 1-3 months, 12 months post-TAVR, and yearly thereafter. Additional unplanned echocardiography was performed when clinically indicated. Echocardiographic data from planned and unplanned echocardiography were prospectively recorded in a database using a standardized case report form.

### Outcomes

The primary outcome was stage 2 (moderate) or 3 (severe) hemodynamic valve deterioration (HVD) occurrence during follow-up, according to VARC-3 definition.^3^ Stage 2 HVD was defined as an increase in mean transvalvular gradient ≥10 ​mmHg resulting in mean gradient ≥20 ​mmHg with a concomitant decrease in effective orifice area (EOA) ≥0.3 ​cm^2^ or ≥25% and/or decrease in Doppler velocity index ≥0.1 or ≥20% compared with echocardiographic assessment performed postprocedure, or new occurrence or increase of ≥1 grade of intraprosthetic aortic regurgitation resulting in ≥moderate AR. Stage 3 HVD was defined as an increase in mean transvalvular gradient ≥20 ​mmHg resulting in a mean gradient ≥30 ​mmHg with a concomitant decrease in EOA ≥0.6 ​cm^2^ or ≥50% and/or decrease in Doppler velocity index ≥0.2 or ≥40% compared with echocardiographic assessment performed postprocedure, or new occurrence, or increase of ≥2 grades, of intraprosthetic aortic regurgitation resulting in severe AR.

Secondary outcomes were the incidence of stage 3 HVD, bioprosthetic valve failure (BVF), all-cause mortality, cardiovascular mortality, aortic valve reintervention, types 2-4 bleeding, ischemic stroke, and myocardial infarction during the follow-up. All events were defined according to VARC-3 consensus.^3^

### Statistical Analysis

Continuous variables were presented as mean ​± ​SD or median and interquartile range (IQR) depending on variable distribution. The distribution normality was assessed with graphical methods for normality. The Student’s t test or the Mann–Whitney/Wilcoxon test was used for continuous variables. Categorical variables were expressed as frequencies and compared with the chi-square test or Fisher exact test when appropriate.

Survival probabilities of events were estimated using the Kaplan–Meier method and the survival curves were compared with the log-rank test. Data on patients who were lost to follow-up were censored at the time of the last contact. The risk of events was assessed with multivariable Cox regression analyses for total death and cardiovascular death, and with Fine and Gray subdistribution hazard models with all-cause death as a competing risk for stage 2 or 3 HVD, stage 3 HVD, BVF, aortic valve reintervention, stroke, myocardial infarction, and types 2-4 bleedings. Each model was adjusted on age, sex, body mass index, chronic kidney disease, prosthesis type, prosthesis size, and valve-in-valve procedure.

To reduce imbalance in baseline and procedural characteristics, the effect of OAC class on events was assessed using 1:1 propensity score-matched population (VKAs vs. DOACs). A nearest-neighbor algorithm without replacement with a caliper width of 0.1 of the SD of the logit of the propensity score was applied. The score included the following covariates: age, date of TAVR (before 2015, 2015-2020, and after 2020), Society of Thoracic Surgeons score, hypertension, chronic kidney disease, prosthesis type, prosthesis size, predilation, postdilation, and valve-in-valve procedure. Covariate balance after matching was assessed using standardized mean differences, with a standardized mean difference <0.10 indicating adequate balance. Graphical assessments were used to confirm overall matching quality.

The evolution of mean gradient and EOA over time in the VKAs and DOACs groups were evaluated using linear mixed-effects models. In these models, the mean gradient and EOA were treated as dependent variables, and OAC class, time, and their interaction were included as fixed effects. A random intercept for each patient was incorporated to account for within-subject correlation due to repeated measurements. Fixed effects were tested using the Satterthwaite approximation for degrees of freedom. The significance of the interaction terms was used to evaluate whether the temporal trajectories of the mean gradient and EOA differed between groups at each time points. In addition, changes in mean transaortic gradient measurements over time (discharge, 1-3 months, 1 year, 2-5 years, and 6-10 years) were evaluated with repeated-measures analyses of variance on patients with echocardiographic measures available at each time point analyzed.

A 2-tailed *p* ​< ​0.05 was considered statistically significant. All statistical analyses were performed using R software (version 4.3.1; R Foundation for Statistical Computing, Vienna, Austria).

## Results

### Patients Characteristics

Overall, 688 patients underwent TAVR during the study period with OAC at discharge, and were alive at 1-year (Supplemental Figure 1). Of these, VKA at discharge was prescribed in 357 patients (52%) and DOAC in 331 (48%). The clinical characteristics of the study population, overall and according to OAC class, are shown in Table 1. Patients with DOAC were older (81 ​± ​7 years vs. 80 ​± ​7 years, *p* = 0.003) and less frequently of female sex (39% vs. 54%, *p* ​< ​0.001), and had a lower Society of Thoracic Surgeons score (4.6% ​± ​3.2% vs. 6.6% ​± ​4.2%, *p* ​< ​0.001), higher rates of hypertension (95% vs. 89%, *p* = 0.006), dyslipidemia (92% vs. 85%, *p* = 0.004), smoking (6% vs. 3%, *p* = 0.034), atrial fibrillation (80% vs. 68%, *p* ​< ​0.001), and a lower prevalence of chronic kidney disease (47% vs. 59%, *p* = 0.002). The use of prosthesis size ≥26 ​mm was higher in patients with DOAC (79% vs. 63%, *p* ​< ​0.001), whereas the rate of predilation was lower (14% vs. 52%). The rates of balloon-expandable valve use, postdilation, and valve-in-valve procedure were similar in both groups (*p* ​> ​0.05 for all).

**Table 1:** Baseline characteristics of the study population, overall and according to anticoagulant type

	Overall population *N* = 688	VKA *N* = 357	DOAC *N* = 331	*p* value	SMD
Age (y)	81 ​± ​7	80 ​± ​7	81 ​± ​7	**0.003**	0.225
Female sex	321 (46.7)	191 (53.5)	130 (39.3)	**<0.001**	0.288
BMI (kg/m^2^)	27.7 ​± ​5.6	27.8 ​± ​5.7	27.7 ​± ​5.5	0.790	0.020
STS score	5.6 ​± ​3.9	6.6 ​± ​4.2	4.6 ​± ​3.2	**<0.001**	0.536
Hypertension	631 (91.7)	317 (88.8)	314 (94.9)	**0.006**	0.123
Dyslipidemia	610 (88.7)	304 (85.2)	306 (92.4)	**0.004**	0.133
Diabetes	255 (37.1)	137 (38.4)	118 (35.6)	0.509	0.056
Smoking	31 (4.6)	12 (3.4)	19 (5.8)	**0.034**	0.200
Coronary artery disease	377 (54.9)	208 (58.3)	169 (51.2)	0.075	0.385
Chronic kidney disease	366 (53.4)	211 (59.1)	155 (47.1)	**0.002**	0.142
OAC indication				**<0.001**	0.242
Atrial fibrillation	509 (74.0)	243 (68.1)	266 (80.4)		
Mechanical heart valve	63 (9.2)	63 (17.6)	0 (0.0)		
Other	150 (21.8)	93 (26.1)	65 (19.6)		
Calcium score	2351 ​± ​1536	2344 ​± ​1593	2357 ​± ​1482	0.916	0.009
Prosthesis type				0.229	0.018
Balloon-expandable valve	504 (73.3)	269 (75.4)	235 (71.0)		
Self-expanding valve	184 (26.7)	88 (24.6)	96 (29.0)		
Prosthesis size ≥26 ​mm	486 (70.7)	224 (62.9)	262 (79.2)	**<0.001**	0.064
Predilation	231 (33.6)	186 (52.1)	45 (13.6)	**<0.001**	0.398
Postdilation	103 (15.0)	66 (18.5)	37 (11.2)	0.229	0.104
Valve-in-valve	65 (9.5)	40 (11.2)	25 (7.6)	0.132	0.096

Values are mean ​± ​SD, or n (%), unless otherwise indicated. Bold values indicate statistically significant *p*-values (*p* < 0.05).

Abbreviations: BMI, body mass index; DOAC, direct oral anticoagulant; OAC, oral anticoagulation; SMD, standardized mean difference; STS, Society of Thoracic Surgeons; VKA, vitamin K antagonist.

### Baseline Characteristics of the Propensity-Score Matched Population

The propensity-score matched population consisted of 132 patients with VKA and 132 patients with DOAC (Supplemental Figure 2). Baseline characteristics of the propensity-matched population are presented in the Table 2. Baseline demographics, main cardiovascular risk factors, and comorbidities did not differ between patients receiving VKA and those receiving DOAC (*p* ​> ​0.05 for all), except for atrial fibrillation, which remained more frequent in DOAC patients (85% vs. 69%, *p* = 0.008). Procedural characteristics did not differ between groups, including similar rates of balloon-expandable valve use, predilation and postdilation use, and valve-in-valve procedure, as well as similar prosthesis size (*p* ​> ​0.05 for all).

**Table 2:** Baseline characteristics of the propensity-score matched population according to anticoagulant type

	Matched population	VKA	DOAC	*p* value	SMD
	*N* = 264	*N* = 132	*N* = 132		
Age – y	80 ​± ​7	80 ​± ​6	80 ​± ​7	0.950	0.009
Female	129 (48.9)	71 (53.8)	58 (43.9)	0.140	0.021
BMI – kg/m^2^	28.0 ​± ​6.1	28.2 ​± ​5.7	27.7 ​± ​6.5	0.520	0.076
STS score	5.3 ​± ​3.4	5.4 ​± ​3.1	5.1 ​± ​3.6	0.402	0.091
Hypertension	244 (92.4)	123 (93.2)	121 (91.7)	0.816	0.035
Dyslipidemia	239 (90.5)	120 (90.9)	119 (90.2)	1.000	0.023
Diabetes	97 (36.7)	54 (40.9)	43 (32.6)	0.202	0.026
Smoking	14 (5.3)	5 (3.8)	9 (6.8)	0.410	0.073
OAC indication				**0.008**	0.345
Atrial fibrillation	203 (76.9)	91 (68.9)	112 (84.8)		
Mechanical heart valve	37 (14.0)	37 (28.0)	0 (0.0)		
Other	47 (17.9)	30 (22.9)	20 (15.2)		
Coronary artery disease	145 (54.9)	74 (56.1)	71 (53.8)	0.805	0.039
Chronic kidney disease	138 (52.3)	74 (56.1)	64 (48.5)	0.267	0.061
Calcium score	2204 ​± ​1515	2169 ​± ​1471	2240 ​± ​1566	0.202	0.048
Prosthesis type				1.000	0.006
Balloon-expandable valve	172 (65.2)	86 (65.2)	86 (65.2)		
Self-expanding valve	92 (34.8)	46 (34.8)	46 (34.8)		
Prosthesis size ≥26 ​mm	197 (74.6)	97 (73.5)	100 (75.8)	0.777	0.027
Predilatation	46 (17.4)	26 (19.7)	20 (15.2)	0.417	0.072
Postdilation	37 (14.0)	19 (14.4)	18 (13.6)	1.000	0.015
Valve-in-valve	26 (9.9)	12 (9.1)	14 (10.6)	0.836	0.008

Values are mean ​± ​SD, or n (%), unless otherwise indicated. Bold values indicate statistically significant *p*-values (*p* < 0.05).

Abbreviations: BMI, body mass index; DOAC, direct oral anticoagulant; OAC, oral anticoagulation; SMD, standardized mean difference; STS, Society of Thoracic Surgeons; VKA, vitamin K antagonist.

### Bioprosthetic Valve Durability According to the Class of Oral Anticoagulation

In the overall population, the median follow-up was 3 years (IQR 2-5). The rates of stage 2 or 3 HVD (DOAC: 16 per 1000 patient-years [95% CI: 9-27], VKA: 16 per 1000 patient-years [95% CI: 10-24], log-rank *p* = 0.972), stage 3 HVD (DOAC: 5 per 1000 patient-years [95% CI: 2-12], VKA: 4 per 1000 patient-years [95% CI: 1-8], log-rank *p* = 0.352), and BVF (DOAC: 16 per 1000 patient-years [95% CI: 9-27], VKA: 13 per 1000 patient-years [95% CI 7-20], log-rank *p* = 0.394) were similar between groups (Table 3). In the competing risk analysis using Fine and Gray subdistribution hazard models with all-cause death as a competing risk, no significant effect of OAC class was observed for stage 2 or 3 HVD (subdistribution hazard ratio [sHR] 1.32, 95% CI: 0.64-2.72, *p* = 0.450), stage 3 HVD (sHR 3.16, 95% CI: 0.60-16.7, *p* = 0.168), and BVF (sHR 1.32, 95% CI: 0.65-2.67, *p* = 0.442).

**Table 3:** Bioprosthetic durability and clinical outcomes according to anticoagulant type in the overall population

Outcomes	VKA *N* = 357	DOAC *N* = 331	sHR (95% CI)	*p* value
Bioprosthetic durability outcomes				
Stage 2 or 3 HVD	16 (10-24)	16 (9-27)	1.32 (0.64-2.72)	0.450
Stage 3 HVD	4 (1-8)	5 (2-12)	3.16 (0.60-16.7)	0.168
BVF	13 (7-20)	16 (9-27)	1.32 (0.65-2.67)	0.442
Clinical outcomes				
Total death	123 (105-143)	90 (72-112)	0.92 (0.70-1.21)	0.568
Cardiovascular death	46 (35-59)	28 (18-41)	0.78 (0.49-1.25)	0.308
Aortic valve reintervention	3 (1-7)	3 (1-9)	1.50 (0.28-8.04)	0.629
Stroke	20 (13-29)	24 (15-36)	1.18 (0.62-2.26)	0.601
Myocardial infarction	7 (3-13)	10 (4-18)	1.66 (0.63-4.37)	0.289
Type 2-4 bleedings	32 (23-44)	30 (19-43)	0.81 (0.50-1.33)	0.410

The incidence of outcomes is presented for each group as events per 1000 patient-y, with 95% CIs.

Multivariable Cox analyses were used to assess the association between OAC class with total death and cardiovascular death. Fine and Gray subdistribution hazard models with all-cause death as a competing risk were used to assess the association between anticoagulation and aortic valve reintervention, stroke, myocardial infarction, type 2-4 bleedings, stage 2 or 3 HVD, stage 3 HVD, and BVF. Each model was adjusted on age, sex, BMI, chronic kidney disease, prosthesis size, prosthesis type, and valve-in-valve procedure. VKA was used as reference in each model.

Abbreviations: BMI, body mass index; BVF, bioprosthetic valve failure; DOAC, direct oral anticoagulant; HVD, hemodynamic valve deterioration; OAC, oral anticoagulation; sHR, subdistribution hazard ratio; VKA, vitamin K antagonist.

**Figure 1:**
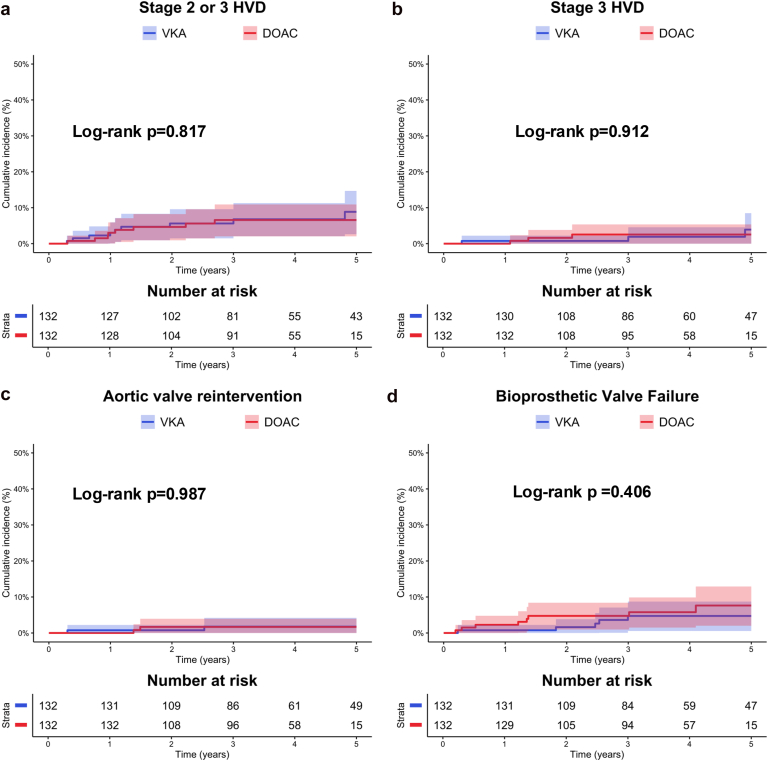
**Long-term hemodynamic valve deterioration, bioprosthetic valve failure, and aortic valve reintervention rate according to anticoagulant class.** Kaplan–Meier graph for the incidence of **(a)** stage 2 or 3 HVD, **(b)** stage 3 HVD, **(c)** aortic valve reintervention, **(d)** and BVF in the VKA and DOAC groups. Blue indicates patients with VKA and red indicates patients with DOAC. Curves were compared using Log-rank test. Abbreviations: BVF, bioprosthetic valve failure; DOAC, direct oral anticoagulant; HVD, hemodynamic valve deterioration; VKA, vitamin K antagonist.

In the propensity-score matched population, the median follow-up was 4 years (IQR 3-5). The rates of stage 2 or 3 HVD (DOAC: 18 per 1000 patient-years [95% CI: 8-35], VKA: 20 per 1000 patient-years [95% CI: 9-38], log-rank *p* = 0.817), stage 3 HVD (DOAC: 7 per 1000 patient-years [95% CI: 1-19], VKA: 6 per 1000 patient-years [95% CI: 1-19], log-rank *p* = 0.912), and BVF (DOAC: 18 per 1000 patient-years (95% CI: 8-35), VKA: 11 per 1000 patient-years (95% CI: 3-25), log-rank *p* = 0.406) did not differ between groups (Table 4, Figure 1). In the competing risk analysis using Fine and Gray subdistribution hazard models with all-cause death as a competing risk, no significant effect of OAC class was observed for stage 2 or 3 HVD (sHR 0.89, 95% CI: 0.35-2.29, *p* = 0.808), stage 3 HVD (sHR 1.06, 95% CI: 0.23-4.97, *p* = 0.938), and BVF (sHR 1.62, 95% CI: 0.53-4.96, *p* = 0.401).

**Table 4:** Bioprosthetic durability and clinical outcomes according to anticoagulant type in the propensity-score matched population

Outcomes	VKA *N* = 132	DOAC *N* = 132	sHR (95% CI)	*p* value
Bioprosthetic durability outcomes				
Stage 2 or 3 HVD	20 (9-38)	18 (8-35)	0.89 (0.35-2.29)	0.808
Stage 3 HVD	6 (1-19)	7 (1-19)	1.06 (0.23-4.97)	0.938
BVF	11 (3-25)	18 (8-35)	1.62 (0.53-4.96)	0.401
Clinical outcomes				
Total death	124 (95-160)	106 (79-140)	0.89 (0.61-1.30)	0.544
Cardiovascular death	53 (34-78)	33 (18-54)	0.65 (0.34-1.24)	0.193
Aortic valve reintervention	4 (1-15)	4 (1-16)	0.97 (0.14-6.82)	0.981
Stroke	13 (5-28)	22 (11-41)	1.87 (0.66-5.27)	0.240
Myocardial infarction	6 (1-19)	13 (5-29)	2.21 (0.54-8.98)	0.273
Major bleedings	31 (17-53)	18 (8-35)	0.56 (0.24-1.34)	0.191

The incidence of outcomes is presented for each group as events per 1000 patient-y, with 95% CIs.

Multivariable Cox analyses were used to assess the association between OAC class with total death and cardiovascular death. Fine and Gray subdistribution hazard models with all-cause death as a competing risk were used to assess the association between anticoagulation and aortic valve reintervention, stroke, myocardial infarction, type 2-4 bleedings, stage 2 or 3 HVD, stage 3 HVD, and BVF. VKA was used as reference in each model.

Abbreviations: BMI, body mass index; BVF, bioprosthetic valve failure; DOAC, direct oral anticoagulant; HVD, hemodynamic valve deterioration; OAC, oral anticoagulation; sHR, subdistribution hazard ratio; VKA, vitamin K antagonist.

**Figure 2:**
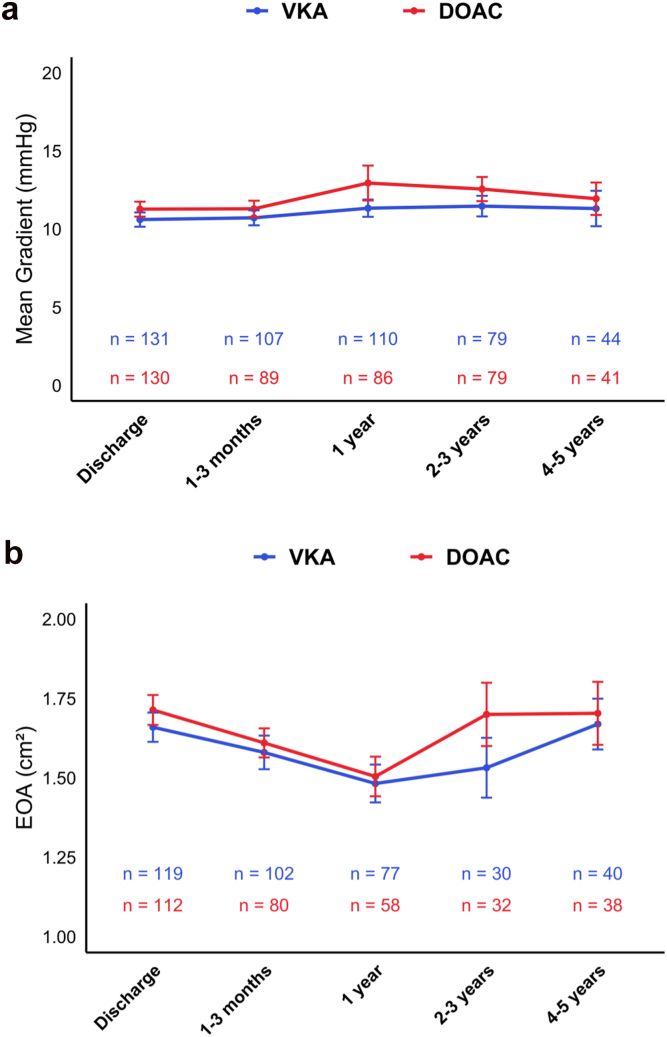
**Evolution of echocardiographic parameters over time according to anticoagulation class.** Evolution of **(a)** mean gradient and **(b)** EOA over time in VKA and DOAC groups. Values are presented as mean ​± ​SD at each time point. Statistical comparisons of trajectories between groups were performed using a linear mixed-effects model with the Satterthwaite approximation. No significant difference was observed at each time point. Abbreviations: DOAC, direct oral anticoagulant; EOA, effective orifice area; VKA, vitamin K antagonist.

**Figure 3:**
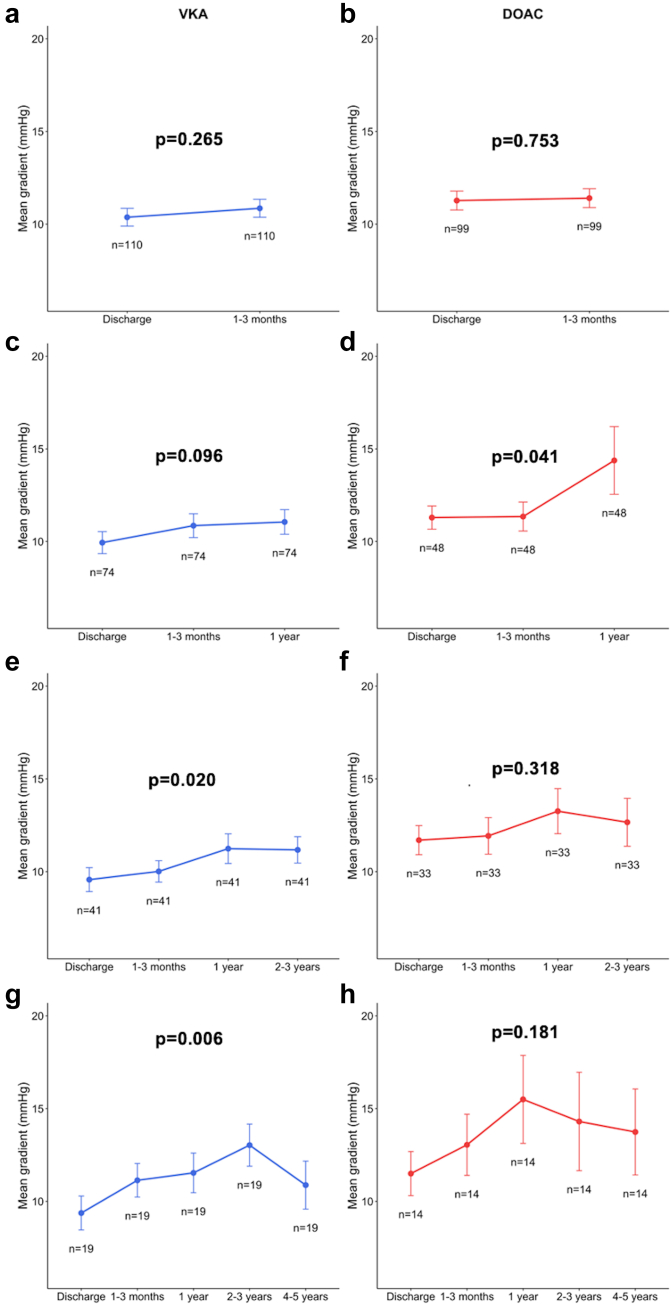
**Progression of mean transaortic gradient over time according to paired repeated-measures analyses.** To evaluate changes in the mean transvalvular gradient over time, repeated-measures analyses were performed. Longitudinal paired comparisons were conducted using repeated-measures ANOVA with patient ID as a within-subject factor to account for intraindividual variability. The temporal comparisons included predefined time points, including discharge, **(a and b)** 1-3 months, **(c and d)** 1 year, and long-term follow-up ([**e** and **f**] 2–3 years and [**g** and **h**] 4-5 years). The cohort was stratified according to anticoagulant type (VKA vs. DOAC), and statistical analyses were conducted independently within each group. For each ANOVA, *p* values were reported to assess the global effect of time on the measured variable. Abbreviations: ANOVA, analysis of variance; DOAC, direct oral anticoagulant; VKA, vitamin K antagonist.

Over time, no significant differences were observed in the evolution of mean gradient and EOA between patients with DOAC and those with VKA, whereas a mild increase of mean gradient was observed in patients on DOAC at 1-year (Figure 2). Paired repeated-measures analyses of variance demonstrated a significant increase of mean transaortic gradient at 1 year in patients with DOAC (*p* = 0.041), and at 2-3 years in patients with VKA (*p* = 0.020) (Figure 3).

**Figure 4:**
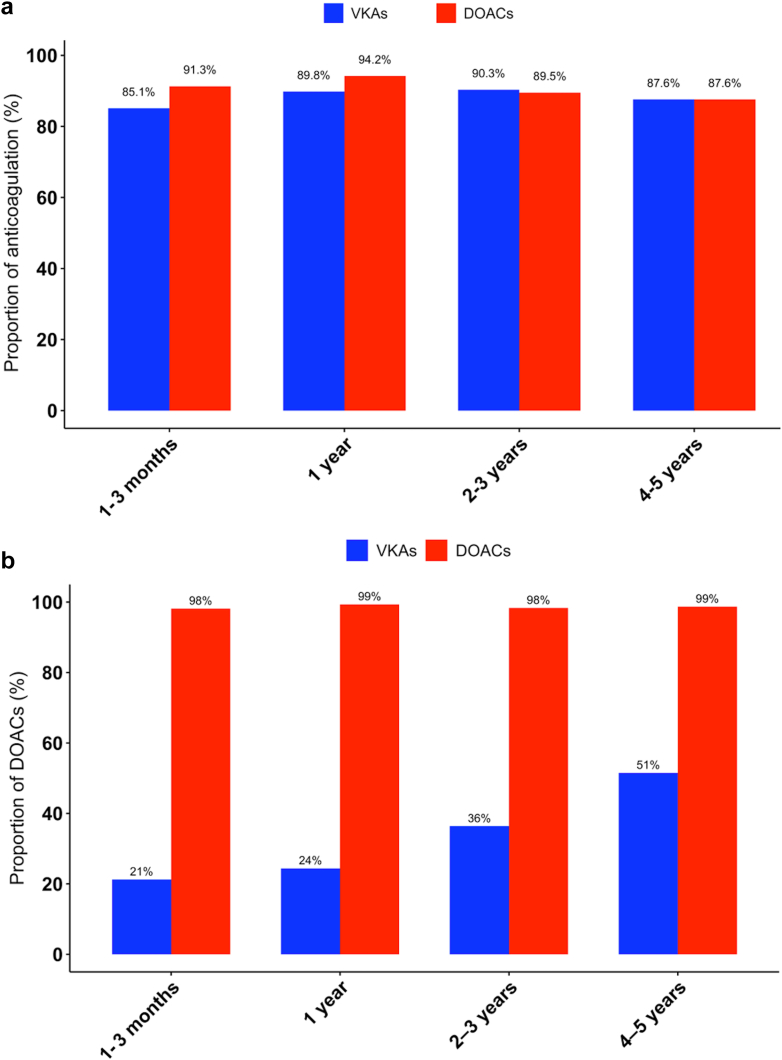
**Evolution of anticoagulation rate and anticoagulation class over time. (a)** Evolution of anticoagulation rate in patients on VKAs and those on DOACs at discharge. **(b)** Evolution of the proportion of patients on DOACs in patients on VKAs and those on DOACs at discharge. Abbreviations: DOAC, direct oral anticoagulant; VKA, vitamin K antagonist.

Anticoagulation rates were overall stable in both groups during the study period (Figure 4). However, although OAC class distribution remained unchanged among patients treated with DOACs, a gradual increase in the proportion of patients initially discharged on VKAs who later switched to DOAC therapy was observed over time (Figure 4).

### Clinical Outcomes Associated with the Class of Oral Anticoagulation

**Figure 5:**
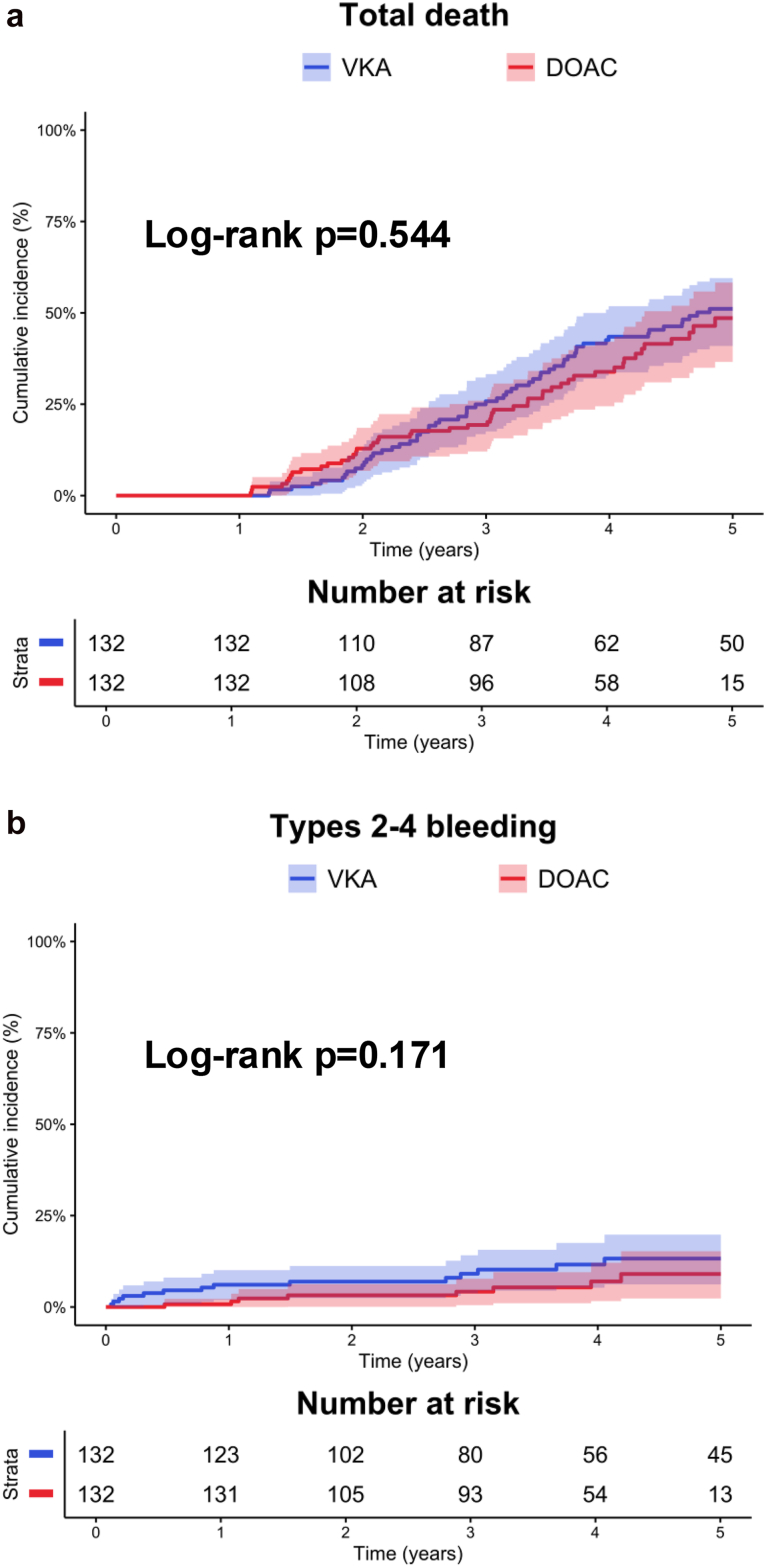
**Clinical events during follow-up according to anticoagulant class in the propensity-matched population.** Kaplan–Meier graphs for the incidence of **(a)** total death and **(b)** VARC-3 types 2-4 bleeding in the VKA and no-DOAC groups. Blue indicates patients with VKA and red indicates patients with DOAC. Curves were compared using log-rank test. Abbreviations: DOAC, direct oral anticoagulant; VARC, Valve Academic Research Consortium; VKA, vitamin K antagonist.

Using Kaplan–Meier and multivariable Cox analysis, OAC class was not associated with a different risk of all-cause death or cardiovascular death, both in the overall population and in the propensity-score matched population (*p* ​> ​0.05 for all, Tables 3, 4, and Figure 5).

Similarly, no differences in the risk of stroke, myocardial infarctions, and types 2-4 bleedings were observed between groups, in both the overall and propensity-score matched populations (*p* ​> ​0.05 for all, Tables 3, 4).

Consistent results were observed in 2 sensitivity analyses on the entire original cohort, also including patients who died within the first year (Supplemental Table 1), and including only patients under OAC for atrial fibrillation (Supplemental Table 2), with no significant differences between VKAs and DOACs in any of the explored outcomes.

## Discussion

The main findings of this study assessing the long-term impact of OAC class on valve durability and clinical outcomes after TAVR are as follows: (1) no significant differences were observed between DOAC and VKA groups in the incidence of stage 2 or 3 HVD, BVF, or aortic valve reintervention; (2) long-term echocardiographic follow-up showed no significant differences in valve hemodynamic parameters between groups; (3) an earlier mild rise in transprosthetic mean gradient was noted among patients receiving DOAC; and (4) clinical outcomes, including mortality and bleeding events, were comparable between patients treated with DOACs and those receiving VKAs.

### Leaflet Thrombosis and Bioprosthetic Valve Dysfunction

Previous studies have highlighted a potential link between leaflet thrombosis and the development of structural valve deterioration. Histopathological analyses suggest that structural valve deterioration is a dynamic, multifactorial process involving thrombosis, endothelialization, and inflammation, which collectively drive leaflet remodeling and ultimately lead to fibrosis and calcification.^4^^,^^17^, ^18^, ^19^ Consistently, observational data have shown an association between leaflet thrombosis and an increased risk of structural valve deterioration at 3-year follow-up.^20^

Although leaflet thrombosis has been reported more frequently after TAVR compared to surgical aortic valve replacement,^21^^,^^22^ the underlying mechanisms remain incompletely understood and may include valve-related mechanical factors^7^ and persistent interaction between the bioprosthesis and residual native aortic valve tissue.^5^^,^^8^ Although not specifically focused on valve-related outcomes, some randomized controlled trials have shown that OAC reduces the risk of leaflet thrombosis.^23^ Altogether, these specific TAVR-related features underscore the importance of optimizing antithrombotic strategies to preserve long-term bioprosthetic valve function.

### Does the Class of Anticoagulant Affect Long-Term Valve Durability?

Despite the suspected role of leaflet thrombosis in bioprosthetic valve dysfunction, the impact of OAC on long-term valve durability after TAVR remains unclear, with conflicting evidence in the literature. Although some studies identified an association between the absence of OAC after TAVR and a higher incidence of HVD during follow-up,^9^, ^10^, ^11^^,^^24^ other reports have challenged this protective association.^25^ A recent registry analysis paradoxically identified OAC use as a predictor of stage 2 or 3 HVD.^12^ One potential explanation for these conflicting findings lies in the differential biological effects of anticoagulant classes. VKAs may promote vascular and valvular calcification by inhibiting matrix Gla protein activation, a key physiological inhibitor of tissue mineralization.^13^, ^14^, ^15^ Prior studies have demonstrated that OAC use, particularly VKAs, may reduce tissue expression of matrix Gla protein, leading to lower circulating levels and increased valvular calcification.^15^^,^^26^ In addition, a recent study demonstrated that aortic stenosis is associated with an increased level of serum Factor Xa activity, and its possible association with disease progression.^16^ This finding may suggest the potential benefits of DOACs targeting Factor Xa. However, no studies to date had specifically investigated whether the class of anticoagulant influences the long-term durability of bioprosthetic valves after TAVR.

To address this gap, we conducted the present analysis to evaluate the association between OAC class (VKAs vs. DOACs) and valve durability over long-term follow-up. At baseline, patients receiving VKAs and those receiving DOACs displayed significant differences in clinical characteristics, largely reflecting differences in comorbidity profiles and procedural date. Indeed, VKAs were more frequently used in patients with chronic kidney disease and were the sole OAC option before the introduction of DOACs in the mid-2010s. Given these imbalances, propensity score matching was used to mitigate potential confounding. After matching, the only remaining difference between the 2 groups was the prevalence of atrial fibrillation. Although atrial fibrillation is common in both groups, VKAs may also be used in patients with mechanical heart valves, a contraindication to DOACs, making full adjustment challenging. After a median follow-up of 4 ​years, no significant differences were observed between DOAC and VKA groups in terms of durability outcomes, including stage 2 or 3 HVD, BVF, or aortic valve reintervention. Consistently, echocardiographic follow-up showed no meaningful differences in valve hemodynamic evolution over time. Interestingly, the expected increase in mean transaortic gradient over time^11^ appeared to occur slightly earlier in patients under DOACs (1 year) compared to those under VKAs (2-3 years). This finding should be interpreted with caution, as it may reflect the lack of adjustment for multiple testing and potential differences in early mortality between treatment groups rather than a true biological effect. Further studies are needed to determine whether this trend reflects a differential efficacy in preventing leaflet thrombosis, possibly related to distinct effects on the contact phase of coagulation, which plays a primary role in bioprosthetic valve thrombosis.

### Clinical Implications

The optimal antithrombotic strategy following TAVR remains an area of active investigation. Given the failure of randomized clinical trials to demonstrate a clear benefit of routine OAC in unselected post-TAVR populations,^27^^,^^28^ alternative strategies involving different anticoagulant agents, dosages, and treatment durations have been explored.

In the context of comparing VKAs and DOACs, the ENVISAGE-TAVI AF trial (Edoxaban versus Standard of Care and Their Effects on Clinical Outcomes in Patients Having Undergone Transcatheter Aortic Valve Implantation–Atrial Fibrillation) is of particular interest. In this study, edoxaban demonstrated noninferiority to VKAs for a composite outcome including death, myocardial infarction, stroke, systemic embolism, valve thrombosis, and major bleeding among TAVR patients with atrial fibrillation.^29^ Although this trial did not evaluate long-term durability outcomes, it provides reassuring evidence regarding the safety and efficacy of DOACs in this population. More recently, the analysis of a large US registry showed that DOACs were associated with a lower risk of bleeding, all-cause mortality, and stroke at 1 ​year after TAVR in comparison with VKA.^30^ A meta-analysis of 5 studies reported no difference in mortality rate between DOACs and VKAs.^31^ Similarly, our study offers reassuring findings by demonstrating no significant differences in bioprosthetic valve durability between patients treated with VKAs versus DOACs. It remains possible that specific subgroups of patients may derive differential benefits from one class of OAC over another, particularly those at higher risk of leaflet thrombosis. These may include patients with anatomical or procedural risk factors such as bioprosthesis underexpansion or asymmetry, small annular size, high body mass index, or chronic kidney disease.^32^ Further studies are needed to identify clinical or procedural profiles that may guide the optimal choice of anticoagulant class following TAVR.

### Limitations

This study has several limitations that should be acknowledged. First, due to its observational design, causal relationships cannot be established from the associations observed. The analysis relied on a retrospective analysis from a prospective registry, which may be subject to inherent selection and information biases. In addition, the study focused on a selected population of patients undergoing TAVR with an indication for OAC and the use of a propensity-score matching approach further reduced the final sample size, potentially limiting statistical power needed to demonstrate differences. Second, the median follow-up duration was 4 ​years. Although this timeframe allows for a long-term assessment of valve durability, it does not exclude the possibility that differences between anticoagulant classes may emerge over a longer follow-up period. Third, all hemodynamic evaluations were performed using transthoracic echocardiography without centralized adjudication by an independent core laboratory. This may have introduced interobserver variability. Fourth, the nonrandomized nature of the study limits the ability to fully isolate the effect of OAC class on bioprosthetic valve durability. In real-world clinical practice, the choice of OAC class may be influenced by multiple factors, including bleeding and ischemic risk, comorbidities, and clinician judgment. Consequently, unmeasured confounding cannot be entirely excluded, despite the use of propensity-score matching. Residual confounding from unrecorded variables may still have influenced the results. The indications for OAC differed between the VKA and DOAC groups, with a higher proportion of patients treated for atrial fibrillation in the DOAC group, whereas VKA use also encompassed patients with mechanical valves and other indications (for instance, history of venous thromboembolism or antiphospholipid syndrome). However, to date, there is no evidence that these different indications may have an impact on bioprosthetic valve durability after TAVR. In addition, our sensitivity analysis restricted to patients with atrial fibrillation provided findings consistent with the main analysis. Finally, anticoagulant class was defined based on the treatment prescribed at hospital discharge, and did not account for changes in anticoagulation status during follow-up. A gradual cross-over between VKAs and DOACs was observed during follow-up, which may have attenuated between-group differences. This trend likely reflects the temporal evolution of anticoagulation practice, characterized by the increasing adoption of DOACs during the study period. Nevertheless, we believe that treatment at discharge reflects a clinically relevant time point, as it corresponds to a critical window during which thrombotic processes involved in valve dysfunction may be initiated.

## Conclusions

In this analysis of a prospective registry with a median follow-up of 4 ​years after TAVR, no significant differences were observed between VKAs and DOACs in terms of stage 2 or 3 HVD, BVF, aortic valve reintervention, or longitudinal hemodynamic valve performance over time. These findings provide reassuring evidence that, in patients with an indication for OAC, the choice of anticoagulant class does not appear to impact bioprosthetic valve durability. Further dedicated studies including patients without OAC as a comparator, longer follow-up, larger population, and randomized designs are warranted to confirm these observations and guide individualized antithrombotic strategies after TAVR.

## Ethics Statement

The study was approved by the local ethics committee, and all patients provided written informed consent for the procedures. The study was conducted in compliance with the Declaration of Helsinki.

## Funding

The authors have no funding to report.

## Disclosure Statement

Antonin Trimaille reports a relationship with Edwards Lifesciences Corporation that includes funding grants. Josep Rodes-Cabau reports a relationship with Edwards Lifesciences Corporation that includes funding grants and speaking and lecture fees and reports a relationship with Medtronic Inc that includes funding grants and speaking and lecture fees.

The other authors had no conflicts to declare.
