# Supplementary material for: Impact of Anticoagulant Class on Long-Term Bioprosthesis Durability Following Transcatheter Aortic Valve Replacement
Source: Struct Heart. 2025 Dec 13;10(2):100786. doi: 10.1016/j.shj.2025.100786 (PMC12810551; doi:10.1016/j.shj.2025.100786)
Supplement: Supplementary data [file mmc1.docx]

**SUPPLEMENTAL MATERIAL**

**Table of Contents:**

- **Supplemental Figure 1.** Flow-chart of the study.
- **Supplementary Figure 2.** Plot of the propensity-score matching.
- **Supplemental Table 1.** Bioprosthetic durability and clinical outcomes according to anticoagulant type in the overall population, including patients who died during the first year (N=885).
- **Supplemental Table 2.** Bioprosthetic durability and clinical outcomes according to anticoagulant type in population with anticoagulation (N=509).

**Supplemental Figure 1.** Flow-chart of the study.

DOAC = direct oral anticoagulant; OAC = oral anticoagulant; TAVR = transcatheter aortic valve replacement; VKA = vitamin-K antagonist.

**Supplemental Figure 2.** Plot of the propensity-score matching.

We performed propensity-score matching of **132 patients** with VKA with **132 patients** with DOAC, according to age, date of TAVR (before 2015, 2015-2020, and after 2020), STS score, hypertension, chronic kidney disease, prosthesis type, prosthesis size, pre-dilation, post-dilation, and valve-in-valve procedure.

Abbreviation: DOAC = direct oral anticoagulant; STS = society of thoracic surgeons; TAVR = transcatheter aortic valve replacement; VKA = vitamin-K antagonist.

**Supplemental Table 1.** **Bioprosthetic durability and clinical outcomes according to anticoagulant type in the overall population, including patients who died during the first year (N=885).**

| **Outcomes** | **VKA**  ***N=431*** | **DOAC**  ***N=454*** | **aHR (95% CI)** | **p value** |
| --- | --- | --- | --- | --- |
| **Bioprosthetic durability outcomes** | | | | |
| Stage 2 or 3 HVD | 15 (10-23) | 15 (8-25) | 1.18 (0.58-2.41) | 0.649 |
| Stage 3 HVD | 4 (1-8) | 5 (2-11) | 2.63 (0.53-13.0) | 0.242 |
| BVF | 14 (9-22) | 23 (14-34) | 1.59 (0.85-2.96) | 0.150 |
| **Clinical outcomes** | | | | |
| Total death | 159 (138-180) | 119 (98-142) | 0.83 (0.66-1.06) | 0.120 |
| Cardiovascular death | 64 (52-79) | 46 (34-61) | 0.77 (0.53-1.11) | 0.161 |
| Aortic valve reintervention | 4 (2-9) | 10 (5-18) | 2.61 (0.84-8.14) | 0.096 |
| Stroke | 21 (14-30) | 24 (15-35) | 1.14 (0.61-2.13) | 0.670 |
| Myocardial infarction | 8 (4-14) | 10 (5-18) | 1.42 (0.58-3.44) | 0.439 |
| Type 2-4 bleedings | 34 (25-46) | 33 (23-46) | 0.88 (0.55-1.41) | 0.611 |

The incidence of outcomes is presented for each group as events per 1,000 patient-years, with 95% confidence intervals.

Multivariable Cox analyses were used to assess the association between OAC class with total death and cardiovascular death. Fine and Gray’s sub-distribution hazard models with all-cause death as a competing risk were used to assess the association between anticoagulation and aortic valve reintervention, stroke, myocardial infarction, type 2-4 bleedings, stage 2 or 3 HVD, stage 3 HVD, and BVF. Each model was adjusted on age, sex, BMI, chronic kidney disease, prosthesis size, prosthesis type, and valve-in-valve procedure. VKA was used as reference in each model.

aHR = adjusted hazard ratio; BMI = body mass index; BVF = bioprosthetic valve failure; CI = confidence interval; DOAC = direct oral anticoagulant; HF = heart failure; HVD = hemodynamic valve deterioration; OAC, oral anticoagulant; VKA = vitamin-k antagonist.

**Supplemental Table 2.** **Bioprosthetic durability and clinical outcomes according to anticoagulant type in population with anticoagulation (N=509).**

| **Outcomes** | **VKA**  ***N=243*** | **DOAC**  ***N=266*** | **aHR (95% CI)** | **p value** |
| --- | --- | --- | --- | --- |
| **Bioprosthetic durability outcomes** | | | | |
| Stage 2 or 3 HVD | 13 (7-23) | 16 (8-28) | 1.57 (0.64-3.82) | 0.323 |
| Stage 3 HVD | 3 (1-9) | 5 (1-13) | 2.51 (0.38-16.5) | 0.341 |
| BVF | 13 (7-23) | 16 (8-27) | 1.33 (0.59-2.97) | 0.490 |
| **Clinical outcomes** | | | | |
| Total death | 130 (108-155) | 93 (73-117) | 0.87 (0.65-1.18) | 0.380 |
| Cardiovascular death | 48 (35-64) | 31 (20-45) | 0.76 (0.46-1.28) | 0.306 |
| Aortic valve reintervention | 3 (1-9) | 3 (0-9) | 2.10 (0.60-7.29) | 0.240 |
| Stroke | 20 (12-32) | 22 (13-35) | 1.11 (0.51-2.42) | 0.791 |
| Myocardial infarction | 6 (2-14) | 10 (5-20) | 1.66 (0.50-5.46) | 0.410 |
| Type 2-4 bleedings | 22 (14-35) | 32 (20-47) | 1.19 (0.65-2.19) | 0.559 |

The incidence of outcomes is presented for each group as events per 1,000 patient-years, with 95% confidence intervals.

Multivariable Cox analyses were used to assess the association between OAC class with total death and cardiovascular death. Fine and Gray’s sub-distribution hazard models with all-cause death as a competing risk were used to assess the association between anticoagulation and aortic valve reintervention, stroke, myocardial infarction, type 2-4 bleedings, stage 2 or 3 HVD, stage 3 HVD, and BVF. Each model was adjusted on age, sex, BMI, chronic kidney disease, prosthesis size, prosthesis type, and valve-in-valve procedure. VKA was used as reference in each model.

aHR = adjusted hazard ratio; BMI = body mass index; BVF = bioprosthetic valve failure; CI = confidence interval; DOAC = direct oral anticoagulant; HF = heart failure; HVD = hemodynamic valve deterioration; OAC, oral anticoagulant; VKA = vitamin-k antagonist.
